# A new environmental public health practice to manage current and future global health challenges through education, training, and capacity building

**DOI:** 10.3389/fpubh.2024.1373490

**Published:** 2024-11-18

**Authors:** Giovanni S. Leonardi, Ariana Zeka, Matthew Ashworth, Catherine Bouland, Helen Crabbe, Raquel Duarte-Davidson, Ruth Ann Etzel, Nia Giuashvili, Özden Gökdemir, Wojciech Hanke, Peter van den Hazel, Paul Jagals, Ejaz Ahmad Khan, Piedad Martin-Olmedo, Joseph Pett, Ekaterine Ruadze, Maria Grazia Santamaria, Jan C. Semenza, Cecilia Sorensen, Sotiris Vardoulakis, Fuyuen Yip, Paolo Lauriola

**Affiliations:** ^1^UKHSA, London, United Kingdom; ^2^LSHTM, London, United Kingdom; ^3^University College London, London, United Kingdom; ^4^Institute of Environmental Science and Research Limited (ESR), Upper Hutt, New Zealand; ^5^The Child Health Research Centre, The University of Queensland, Brisbane, QLD, Australia; ^6^Université Libre de Bruxelles (ULB)—Ecole de Santé Publique, Brussels, Belgium; ^7^Exeter University, Exeter, United Kingdom; ^8^Milken Institute School of Public Health, The George Washington University, Washington, DC, United States; ^9^National Center for Disease Control and Public Health, Tblisi, Georgia; ^10^Faculty of Medicine, Izmir University of Economics, İzmir, Türkiye; ^11^Nofer Institute of Occupational Medicine, Lodz, Poland; ^12^International Network on Children’s Health, Environment and Safety (INCHES), Ellecom, Netherlands; ^13^WHO Collaborating Centre for Children’s Health and Environment, The University of Queensland, Brisbane, QLD, Australia; ^14^Health Services Academy, Islamabad, Pakistan; ^15^Andalusian School of Public Health, Granada, Spain; ^16^Ibs. GRANADA, Granada, Spain; ^17^IHR Strengthening Project, UKHSA, Bangkok, Thailand; ^18^ASL Foggia, Foggia, Italy; ^19^Department of Public Health and Clinical Medicine, Umeå University, Umeå, Sweden; ^20^Global Consortium on Climate and Health Education at Columbia University, New York, NY, United States; ^21^HEAL Global Research Centre, Health Research Institute, University of Canberra, Bruce, ACT, Australia; ^22^US-CDC, Atlanta, GA, United States; ^23^International Society Doctors for the Environment (ISDE), Rete Italiana Medici Sentinella per l’Ambiente (RIMSA), Arezzo, Italy

**Keywords:** environmental health, public health, ecology, professional training, education, ecological sustainability, environmental change, ecological public health

## Abstract

Unsustainable globalisation of economic activities, lifestyles and social structures has contributed to environmental degradation, posing major threats to human health at the local and global levels. All these problems including climate change, pollution, and biodiversity loss represent challenges that are unlikely to be met with existing approaches, capabilities and tools. This article acknowledges the need for well-prepared practitioners from many walks of life to contribute to environmental public health (EPH) functions thus strengthening society’s capacity and capability to respond effectively and in a timely manner to such complex situations and multiple challenges. It envisions a new EPH practice addressing questions on: Why do this? What needs to be addressed? Who will do it? How can it be implemented? This article focuses on the main challenging EPH issues worldwide and how they could be addressed using a conceptual framework for training. A companion article shows how they have been tackled in practice, providing ideas and experiences.

## 1 Challenges for environmental public health

Population health problems are complex, as they are determined by environmental, social, economic, and political factors at the local through global levels [e.g., climate crisis (1), obesity (2)]. This requires an integrated and holistic approach for public health science and policy formulation which is also reflected in the WHO social determinants of health framework (3, 4). This paradigm takes into account the distal and structural determinants of health (e.g., economic or employment policies, access to quality housing, healthy food and sustainable transport) as fundamental in determining the unequal distribution of the proximal risk factors (e.g., air pollution, unhealthy diet, sedentary lifestyle, and smoking), and the health status and disease within and across populations (5). Furthermore, policies and actions outside the health sector (e.g., traffic regulations, urban planning and availability of green spaces, obesogenic environment, and food quality) (6) are known to contribute to adverse health outcomes.

The globalisation of economic activities, lifestyles and social structures have contributed to both local and global environmental degradation and change. The collective consequence of the greenhouse gases emissions by individual societies worldwide is perhaps the best-known example of this. At the same time, greater awareness of environmental impacts has been enabled by global linkages, thus resulting in changes at the local (e.g., urban planning) and at the global level (e.g., international agreements), starting with the recognition by the Rio Earth Summit 1992 (6). Several countries have integrated in their legislation the notion of sustainability, the right to health and the right to a healthy environment, and more recently, the UN declared access to a healthy environment a Human Right (7).

The current ‘triple planetary crisis’ including climate change, environmental pollution, and biodiversity loss, pose major threats to human health both at the local and global level. The impact of these complex challenges is affecting populations worldwide, often unequally distributed with many populations having less or no access to adequate housing, health services, and basic resources such as clean air, water, energy, and healthy foods. These challenges require new approaches and tools in addressing impacts of complex drivers and exposures (8), incorporating concepts such as cumulative impacts of environmental decisions (9), with multiple dimensions from social indicators, living and working conditions, behavioural indicators, or infrastructure such as green space and transport. Environmental Public Health (EPH) (10) is the discipline that addresses and studies such complexity from a multilevel perspective and is increasingly implemented by public health services and environmental protection agencies.

Therefore, the challenge of making a skills transition is urgent, and the poor integration between skills initiatives and the needs of the green transition needs addressing (11).

This article acknowledges the need for well-prepared practitioners from many walks of life to contribute to environmental public health functions thus strengthening society’s capacity and capability to respond effectively to such complex situations and multiple challenges. In particular, it will focus on the capacity and capability building required to plan and implement renewed roles for EPH practitioners at local, national, and potentially international levels. The idea for this new role emerged via experiences of multi-disciplinary work beyond the health professions, with practitioners from many sectors including agriculture, town and country planning, engineering, energy, and transport. This type of work often contributed to or accompanied multi-disciplinary interventions (chemical, heat, and flood-related) that supported ‘primordial prevention’. The framework for achieving this objective revolves around two interconnected concepts: the ‘Common Home’, representing the Earth as a shared habitat for all populations, and the ‘Common House’, symbolising a collaborative space where diverse disciplines come together, from the local level to global.

## 2 Paradigms for addressing new challenges in EPH

Several frameworks facilitate the task to reinforce EPH services. The Driving force-Pressure-State-Exposure-Effect-Action (DPSEEA) framework approach, initially promoted by WHO, has been further adapted to seek population wellbeing within an ecosystem perspective (e-DPSEEA) (12). The framework allows practitioners to describe, heuristically, a logical conceptual sequence of events that leads to an environmental health problem (13). It is intended to highlight the important links between different aspects of development, environment, and health and to help identify effective policies and actions to control and prevent health effects (14). Adopting this framework requires multi-disciplinarily education and training theories and tools, including knowledge of exposure analysis, environmental epidemiology, and health impact assessment tools.

The Ecological Public Health approach can help address the challenges faced by public health today, by its aims to integrate complexity, multiple interactions and change of societal systems. By doing so, the approach has the ability to understand the ecosystem processes and the system as a whole, and the way it determines population health. Ecological public health provides a framework for considering a holistic approach from public health science to public health actions (10). The ecological approach to public health has been compared to four different conceptual models recognisable in public health: health-environmental, biomedical, behaviouristic-social, and technological-economic. Each of these models has led to successes but has also been characterised by limitations. Both, the successes and limitations have been evaluated in detail elsewhere (15), and within the constraints of the present article one can conclude that the ecological public health model complements and has the potential to integrate other models of public health practice. Prevention services that ignore the conclusions identified in risk assessments at the local community and global level risk being seriously insufficient. Building prevention services based on a shared culture, values and behaviours would make the public health practice work more valid and solid. This is illustrated for example in the case of obesity by recognising that interventions to reduce this problem may be identified more holistically if social and cultural dimensions are considered as deserving to be addressed in themselves alongside physical and biological ones (2).

The ‘Health in All Policies’ (HiAP) approach, promoted by WHO and adopted by the European Union (EU) in 2006 (16), recommends collaborations through development of intersectoral policy and governance (17). HiAP outlines the adoption of a political overarching vision for a healthier and more sustainable society where all public policy areas can have, directly or indirectly, an impact on health and socioeconomic equity (18). It emphasises the consequences of public policies on health systems, determinants of health and wellbeing, and it enhances the accountability of policymakers for health impacts at all levels of policymaking (19).

However, the ability to respond to such conceptual frameworks for EPH service or the policy recommendations, will be highly dependent on the local context, history, culture, organisational arrangements, capacities and needs, and on who can afford to implement these (20). This recalls the distinction between global and local, as acting within the dimensions of space and time, which is an essential issue for EPH. During the COVID-19 pandemic (21), all the local and global actions, were inextricably linked, being essential to manage the pandemic in many parts of the world.

Currently, there is not an international reference for improving capacity and capability building of EPH functions at national levels. The present form of the WHO-International Health Regulations (22) does not acknowledge environmental factors as determinants of health. Public participation is also an essential force in promoting environmental health quality (23). Supportive policies, regulations, and planning tools would encourage citizens to engage in climate change adaptation and local environmentally friendly planning, and effective and meaningful participation is crucial to ensuring socially just policies (24).

The globalisation of economic activities, social structures, and lifestyle has contributed to both local and global environmental degradation and change. The refore, the role of democracy in pursuing health as the ‘common good’ (25) may counterbalance the powerful financial and economic pressures on governments. An example is the Erasmus Generation in Europe a term that describes young people who participate in mobility programmes, giving them the opportunity to spend part of their studies abroad, do internships, or work. Through their cultural openness and interconnectedness, they show ability to contribute to building knowledge and the capacity to respond to climate change (26). The EU Erasmus programme contributes to a share of knowledge and understanding, democratisation of accessibility to science and to building a generational capacity for understanding common good. Although a regional only example, the model offers excellent insight on the benefits in lack of boundaries in knowledge transfer and the cultural shift in what community responsibility and response means.

The concept of Planetary Health focuses on analysing and addressing the impacts of human disruptions to Earth’s natural systems on human health and all life on Earth (27). Planetary health education across all levels and disciplines will allow transdisciplinary and mutually reinforcing actions to protect and restore planetary health and achieve the Sustainable Development Goals (SDGs) (28).^1^

A simplified set of essential public health functions may be defined based on experience from national public health institutes internationally: (i) preparedness and response; (ii) public health surveillance; (iii) evidence gathering/impact analysis/evaluation of interventions; (iv) effective communication with multiple stakeholders; and (v) advocacy for public health (29). These may be adapted to provide Environmental Public Health (EPH) functions. Table 1 shows how the key EPH functions are mapped and motivated with relevance to each SDG. Some examples of such functions are provided in Reid, 2015 (30) and WHO, 2018 (31). The current status of implementation of these functions varies by country, and so does the role of agencies in delivering them (see section 5). Unless success is built for Goal 13 ‘CIimate Actions’, all the other SDGs will become unattainable (32, 33). Since 2016, the UN has moved from giving highest priority to economic development in a country, to geographic groupings as the highest level of aggregation for SDG statistics (34, 35). This is consistent with the priority advocated in the present paper to consider a country’s history and culture (36) as essential context for designing ecologically sustainable communities.

**Table 1:** Relationship between sustainable development goals and environmental public health functions.^b^

		Environmental public health function				
Sustainable development goal		Preparedness and response	Environmental public health tracking/surveillance	Evidence gathering/impact analysis/interventions	Effective communication	Advocacy
Goal 1	No poverty		Y	Y	Y	Y
Goal 2	Zero hunger	Y	Y	Y	Y	Y
Goal 3	Good health and well-being	Y	Y	Y	Y	Y
Goal 4	Quality education		Y	Y	Y	Y
Goal 5	Gender equality		Y	Y	Y	Y
Goal 6	Clean water and sanitation	Y	Y	Y	Y	Y
Goal 7	Affordable and clean energy		Y	Y	Y	Y
Goal 8	Decent work and economic growth		Y	Y	Y	Y
Goal 9	Industry, innovation and infrastructure		Y	Y		Y
Goal 10	Reduced inequalities		Y	Y		Y
Goal 11	Sustainable cities and communities		Y	Y		
Goal 12	Responsible consumption and production		Y	Y	Y	Y
Goal 13	Climate action	Y	Y	Y	Y	Y
Goal 14	Life below water		Y	Y	Y	
Goal 15	Life on land		Y	Y	Y	
Goal 16	Peace, justice and strong institutions		Y	Y	Y	Y
Goal 17	Partnerships for the Goals		Y	Y		Y

Note: Y= “Yes, a relationship between a SDG goal and an EPH function may be recognised.” ^b^Preparedness and response: Preparedness—making arrangements, creating and testing plans, training, educating and sharing information to prepare communities should an emergency eventuate. These are also ACTIONS and they are happening all the time. Response—the assistance and intervention during or immediately after an emergency. (https://resilience.acoss.org.au/the-six-steps/leading-resilience/emergency-management-prevention-preparedness-response-recovery). Environmental public health tracking/surveillance: systematic, ongoing collection and analysis of information related to disease and environment, and its dissemination to individuals and institutions. (See Annex). Evidence gathering /impact analysis/interventions: are designed to unearth the ‘unexpected’ negative effects of a change on an organisation. It provides a structured approach for looking at a proposed change, so that you can identify as many of the negative impacts or consequences of the change as possible (https://www.mindtools.com/axt4kh3/impact-analysis). Effective communication: is a process of exchanging ideas, thoughts, knowledge and information such that the purpose or intention is fulfilled in the best possible manner. In simple words, it is nothing but the presentation of views by the sender in a way best understood by the receiver (https://theinvestorsbook.com/effective-communication.html). Advocacy: activities related to ensuring access to care, navigating the system, mobilising resources, addressing health inequities, influencing health policy and creating system change are known as health advocacy. (https://pubmed.ncbi.nlm.nih.gov/27866451/#:~:text=In%20the%20medical%20profession%2C%20activities,are%20known%20as%20health%20advocacy).

## 3 The concept of the new EPH practice

### 3.1 Interdomain partnerships and collaboration

This section introduces a novel environmental public health model for healthcare leaders to comprehend the intricacies of climate change, pollution and Planetary-One Health. Having a deep understanding of the scientific aspects and potential solutions, whilst acknowledging the remaining uncertainties, may be highly beneficial. Health practitioners at various levels have shown it is possible to overcome unique challenges influenced by geography, population vulnerabilities, and socio-economic contexts by evaluating emerging knowledge, from individual health guidance to community-wide adaptation planning. A comprehensive health system response that merges medicine and public health, could help address issues at the intersection of the environment and climate. With the unfolding climate crisis, healthcare professionals are encouraged to take a coordinated, proactive measures encompassing primordial, primary, secondary, and tertiary health prevention (37).

A pivotal experience unfolded in Georgia through a Twinning project, a European Union instrument for institutional cooperation between Public Administrations of EU Member States and of beneficiary or partner countries,^2^ involving institutions from Italy, Poland, and the United Kingdom. The project aimed to transfer EU environmental health regulations (38) to the Georgian National Centre for Disease Control (NCDC), focusing on laws, organisational enhancement, and workforce competence. This provided a context for exploration of novel approaches to public health interventions addressing environmental factors for health across a country. A pressing concern emerged: a surge in lead (Pb) poisoning cases across Georgia. Alongside a monitoring programme, preventive measures were deployed and led to a notable reduction in children’s blood lead levels. This progress was achieved in a multi-disciplinary collaboration including health care practitioners, epidemiologists, and natural scientists across existing institutional divisions, and indicates the feasibility of co-ordinated effort to face a novel environmental challenge (39).

### 3.2 What is the new EPH practice?

An approach for identifying essential competencies in public health is extended to pinpoint valued skills and competencies by current employers (40).

The new competencies for EPH may be applied to the ‘three domains’ of existing public health practice, described in terms of three interrelated but distinct dimensions: (1) health promotion, which draws heavily on the local government roots of the profession, socioeconomic influences and health promotion, and tackling the underlying determinants of health; (2) health protection, which incorporates communicable disease control; environmental, chemical, radiation and nuclear threats; and occupational health; (3) health service quality improvement, which incorporates healthcare systems, service quality, evidence-based practice, clinical effectiveness and health economics (41). This provides a robust operational framework that includes the areas of practice, the services to be delivered, and the roles and responsibilities of those delivering them. This is particularly important in describing the core skills, knowledge, and competencies needed so that the respective workforce can carry out their respective roles. As such, this framework has the potential for adaptation to underpin educational and training provisions (42).

The overlap of the three domains of public health practice also helps inform the development of education and training addressing planetary health from the perspective of environmental public health service. In this context, skills and competencies related to dealing with health impacts of the climate crisis are integrated in the curriculum of public health schools and are now a required competency for a health practitioner in some locations (43).

The skills mentioned apply to the advancement of public health across all fields, extending beyond healthcare services. This approach is justified as healthcare systems are responsible, as estimated previously, for approximately 4% of global greenhouse gas emissions. Thus, broad societal changes, and not purely clinical health care of individuals, are required to safeguard public health. There is a growing call for closer collaboration between clinical medicine and public health. The COVID-19 crisis underscored the pivotal role of primary and community healthcare (P&CHC) in both short-term and long-term healthcare. It highlighted the necessity for P&CHC to collaborate in contact tracing efforts, aligning with various healthcare organisations including local and national public health institutions, as well as community-level groups and health workers.

### 3.3 Scoping the tiers/levels of the new EPH practice

Recognising the central role of the development of social change towards sustainability (44), the role of public health education and training is essential. The public health workforce can be defined as ‘a diverse workforce whose main responsibility is the provision of the main health activities for the public, regardless of their organisational basis’, emphasising the broad and diverse nature of public health. Public health increasingly includes the role of the ‘wider’ workforce: people who are indirectly involved in activities but whose work can contribute to improving population health (45).

Practitioners of that broader public health can be divided into three groups: (1) public health specialists; (2) people indirectly involved in public health activities through their work; and (3) people who should be aware of the implications for public health in their professional life (46). To this end, practitioners of all three groups require knowledge and skills in similar fields but on a different level. This holds for general public health, and similar considerations can be made first and foremost for environmental public health (EPH). The evaluation of which disciplines and knowledge are relevant to forming a category of professionals could be conducted by scientific societies and professionals to whom this task was relegated by the State or other parts of civil society (47).

Practitioners in each of these group share the same mission to develop capacity towards achievement of the SDGs as outlined in section 1.3, and according to the roles outlined in section 5. The proposal for this role is considered within the context of community, history, culture, economic and social dimensions. The practitioner originates from the community and works across the disciplines, knowledge and resources available. The proposal, based on experiences from many communities, also offers integration of knowledge beyond the community boundaries, for regional and global sharing of such experiences. Also, the values of justice, culture, and relationships highlighted by consensus statements of Indigenous communities on the theme of planetary health are best served by choice of governance that are inclusive of representatives of indigenous communities proposing their specific perspectives, methods, and topics (36, 48). This is expected to benefit all communities and individuals within them, as it communicates that the key step towards ecological sustainability is not the provision of a new institutional service, but the recognition of the value, wealth, and health already present in each community and individual, and that can be re-oriented to take a new course when faced with challenges related to climate change. In any case, stakeholder engagement needs to consider those affected by climate and environmental change and set out a clear strategy for communication and engagement, ideally extending to a role in the design and monitoring of any research or intervention.

### 3.4 How and why does the public health practitioner advocate and assume custodianship?

Health status is a synthesis or ‘super-indicator’ of the social impact of multiple influences from all sectors of human decision and activity. Therefore, health integrates all other activities (49). Indicators are key to awareness of changes in health status in time and space, and to inform activities to take custody of public health by interventions addressing preventable factors.

Acceptance of the value of health information as a series of indicators of relevance for decisions beyond the health sector is key to the development of ecologically sustainable communities.

In the context of addressing the climate crisis, practical initiatives exemplified by the efforts of *Santé Publique France* and the International Association of National Public Health Institutes (IANPHI) highlight the use of health as a catalyst for action (50). Recent discussions within WHO Europe underscore the pressing challenge posed by the ‘triple crisis’, arising from the interconnected issues of climate change, environmental pollution, and biodiversity loss (51).

Within this framework, a public health practitioner actively engaged in EPH, whether within the healthcare sector or other sectors, can play a crucial role in spearheading the development, design, analysis, and evaluation of systems aimed at incorporating ecological sustainability into various programmes and projects. The EPH practitioner may assume diverse roles, from advocating and motivating cross-sectoral collaborative efforts to challenging the status quo. Communication with those impacted by environmental issues is key as part of consultation and possible collaboration with agencies and groups responsible or affected. Such a role might initially be met with resistance, but it has the potential to prompt alternative, environmentally resilient infrastructure and service solutions.

Simultaneously, any healthcare practitioner, including those in clinical and social care settings unrelated to prevention services, can act as an advocate for the inclusion of health and social assessments in infrastructure and service planning. When such plans are positioned as steps towards sustainability, the consideration of health, well-being, and social impacts offers a tangible contribution to intervention selection, complementing the customary technical and financial criteria mandated by law and tradition.

## 4 How does the new environmental public health practice operate?

### 4.1 Practising environmental public health within the health work force

#### 4.1.1 EPH as a public health task

First, a few assumptions are made regarding public health:

1. Protection and promotion of healthy lives in their social, economic, and environmental context.
2. The overall approach is analytical and systemic at the same time, i.e., observing reality from two different but complementary perspectives: the analytical (reductionist) perspective and the systemic one (since the problems are all interconnected and interdependent), each of which makes use of a broad heritage of methods, knowledge and skills.
3. An interdisciplinarity and cooperative approach is required. Specialisation always must follow the integration of knowledge and cooperation between the parties, so that exaggerated attention to detail does not produce deleterious effects on the general economy of the system. To be successful, therefore, it is necessary to develop one’s professional profile but also to share knowledge, learn from each other and channel creativity towards cooperation and the realisation of common and shared objectives.

The public health workforce encompasses a diverse collective responsible for executing essential public health tasks, irrespective of their institutional affiliations. These workforce members fall into three primary categories (as outlined in section 4.3) and require varied levels of knowledge and skills in related areas. This principle holds true for public health in general and must be transferred to environmental public health. The determination of the relevant disciplines and expertise needed to categorise professionals can be delegated to scientific and professional societies, either by the State or other civil society segments.

For example, the Netherlands, at the request of several ministries, created a category of environmental health specialists characterised as public health physicians with additional training in toxicology, environmental sciences and epidemiology (52). In England, the Faculty of Public Health, comprised of specialised public health professionals, including physicians and other experts, established the necessary competencies in environmental public health (52, 53). Besides general organisational competencies in research, teaching, and service management, five areas of specialised expertise are identified for public health practitioners in this field (54): toxicology, natural sciences, environmental epidemiology, risk assessment, and environmental public health.

#### 4.1.2 EPH as a task for clinical health care workers

Second, EPH has a role within clinical health care professionals. Healthcare primarily concentrates on diagnosis and treatment. Practical knowledge, gained from everyday hands-on experiences, complements procedural knowledge, which deals with how to perform specific tasks in clinical, public, environmental health, or management. Health professionals can drive social and policy change (55) as they are generally highly trusted (56) and have influence at all levels of society. With trust comes the responsibility to influence wisely and lead effectively, which requires collaborative engagement beyond individual actions (47), thus ‘Health professionals will be called on to engage as humble, informed, and trusted partners in the collective, boundary-crossing effort of transforming practises and structures to better sustain the health and wellbeing of all life, including our own’ (57).

Training of professionals in sustainable development and the green economy with a focus on public health management and risk assessment would be beneficial to protection and promotion of healthy lives by prevention of causes of ill-health. They should also be familiar with urban health and pollution-related issues to address various diseases and ensure healthier urban environments (58).

#### 4.1.3 EPH as a task for practitioners in many disciplines

Third, EPH covers contributions from both public health and clinical health care (15). Typically, clinical and public health knowledge often do not integrate knowledge on the health determinants related to environmental health, which include population impacts of pollution, biodiversity loss or climate change—therefore defining boundaries between the two services (59). It is encouraged for medical and health knowledge to recognise one identical set of values and criteria with prevention as an essential focus for clinicians, healthcare practitioners, and public health professionals (60).

Public health services worldwide are increasingly fostering interventions that aim to protect and promote health and the environment (61) as described in Table 2.

**Table 2:** Public health services.

Services of health protection
Supply of potable water Control and safe use of foods and medicines Control of air quality Proper disposal of wastes Fluoridation and oral hygiene Control of radiation and toxic agents Occupational safety Injury prevention Surveillance and control of infectious diseases Urban planning
Individualised services for health promotion
Encourage physical activity and exercise Encourage proper nutrition Encourage personal and household hygiene Encourage respect for others
Collective services of health promotion
Advocacy and public policies Control the use of tobacco, alcohol and other drugs Providing decent and sanitary housing Standards for urban development Green zones Walkways and pedestrian zones Bikeways Development of social capital The organisation of the community Civic culture—Respect The organisation of the community
Preventive medical services
Family planning Control of pregnancy, childbirth and puerperium Growth and development Immunizations Prevention of teen pregnancies Screening and monitoring of cases

Source: Echeverry (62), modified.

There is growing recognition that health care professionals require further training about EPH. The Association for Medical Education in Europe for example, emphasises the importance of equipping health professionals with the knowledge, skills, and values that promote sustainable health, and advocate for environmental and social change whilst protecting the planet (47).

Health care professionals are encouraged to engage with environmental concerns both in their role as clinicians, environmental and public health practitioners, regardless of the national organisation. This applies to undergraduate students, as declared by teachers (63, 64) and students eager to stimulate their institutions in this direction (65). It is also relevant to clinicians in training and those already practising, particularly those defined as Family Doctors (FDs) (21) and Family Paediatricians (FPs) (66). EPH training of physicians (as well as for non-physicians, see below) should include knowledge and skills in recognising, diagnosing and treating health problems caused by environmental risk factors (ERFs) (see Table 3).

**Table 3:** Fields of knowledge relevant to disease prevention, with particular attention to key areas for environmental health.

Prevention and control of injuries	Predictive modelling of injury scenarios Contingency and logistics management Perception of injuries and psychology Economics of injuries and risk
Water quality	Water and wastewater chemistry Applied hydrology Hydrogeology Marine science Hydromechanics Technologies for water supply and water treatment Agriculture, industry and energy management
Air quality	Climatology Industrial management and energy production Meteorology Atmospheric chemistry Pollutants behaviour Atmospheric and climatological modelling Monitoring and modelling
Food quality and safety	Economics of agricultural management Veterinary Sciences Soil Sciences Food production technology Risk analysis systems Food health promotion Biotechnologies and genetic modification technologies
Waste management and soil pollution	Solid and liquid waste management Soil science Management and rehabilitation of contaminated land Waste prevention management
Human ecology and housing	Building management Housing conception and use Territory planning Rural management Architecture Urban planning Construction and housing sciences
Worker’s health	Ergonomics Job security Environmental Protection Engineering technology Work hygiene Bioengineering technology
Energy	Modelling and forecasting energy consumption Long-distance monitoring and remote sensing techniques Geographic information systems Energy transport Energy production and consumption
Transport management	Economics of transport and logistics Transport modelling Engine engineering Behavioural studies on transport Road safety studies
Land use planning	Urban and rural development plans Management of open spaces Nature conservation and wildlife protection Management of the contaminated territory Agricultural management Management of natural resources and energy
Agriculture and fish production	Plant and crop science Animal husbandry Veterinary science Chemical and pesticide safety Marine and fisheries sciences One Health approach
Ionising and non-ionising radiation	Techniques for monitoring and radiation protection of the natural background Safety audit of nuclear power plants Management of nuclear waste Radiation monitoring Predictive modelling techniques Remediation techniques for contaminated sites Epidemiological techniques applied to the study of exposure to non-ionising radiation sources
Noise control	Noise exposure assessment techniques Study of noise-induced disturbance Community-wide noise assessment
Tourism and recreation	Bathing water quality Control of recreational facilities Applied ecology Littoral and estuarine sciences
Control of disease vectors	Entomology Parasitology Applied zoology Infectious disease control techniques One Health approach

Source: Leonardi et al. (15).

### 4.2 Practising environmental public health outside of the health work force

Environmental health, environmental public health and prevention are matters that involve many disciplines and competencies.

Over 70 professional categories relevant to environmental health in Europe were identified in a review published in 1998, including academics, medical specialists, environmental scientists (e.g., epidemiologists, natural scientists, social scientists and experts in occupational hygiene) and professionals (such as environmental health workers, technicians, and architects) (67). Yet, few of these professions have increased their involvement in environmental health and prevention in the past two decades. Prevention requires a continuous updating of knowledge in multiple disciplines to be translated into actions to protect and promote health. That knowledge concerns particularly the environmental factors (Table 3).

Close collaboration between a wide range of thematic areas and scientific disciplines may be helpful given the great diversity of professional categories involved in prevention in general and environmental health in particular. There also is a clear need for intersectoral collaboration, as recognised by the WHO with the ‘Health in All Policies’ approach (68). In practice, a limited number of agencies and groups may be required for any specific project or programme, and consultation with scientific and professional societies may allow to identify appropriate individuals.

Attention must also be paid to those working in environmental management/sustainability professions to enhance their knowledge of health/PH/EPH—focusing on the health impacts of their efforts, to align to the EPH goals (57).

### 4.3 How do these roles integrate, collaborate, and co-function?

The overarching objective of this approach is to realise the full health potential for all individuals, with a dual focus: firstly, to promote and safeguard health within the context of the environment and life transitions, and secondly, to curtail the prevalence of major diseases and injuries whilst mitigating their associated suffering.

Its ethical foundation rests on three fundamental dimensions and principles:

1. Healthy Environment as a Fundamental Human Right: Recognising access to a healthy environment as an inherent human right (7).
2. Equity in Health and Solidarity: Emphasising health equity and collective responsibility (69).
3. Participation and Responsibility: Promoting active involvement and shared responsibility in health development (70, 71).

Despite the extensive promotion of action strategies grounded in scientific, economic, social, and political sustainability, their full implementation remains unrealized.

The earliest codification of intersectoral action is exemplified in Harris et al. (72), where elements of the health sector and other sectors, including environment, transportation, energy, urban planning, and social care, collaboratively address health issues to achieve more effective, efficient, and sustainable outcomes. Such intersectoral action necessitates involvement of policy authorities and national and local practitioners, being multidisciplinary, transdisciplinary, and inclusive of various agencies and stakeholders. It is an ongoing dynamic process.

The importance of improving the relationship between public and private research and science was confirmed in a WHO conference (73).

Preventive services are encouraged to integrate scientific advancements through collaborations with entities such as civil protection and public health institutions. To enhance prevention services, individuals from diverse educational backgrounds may participate, with training programmes blending scientific and professional competencies. This can be achieved through curriculum development, integration, and cross-institutional training experiences. Training duration may vary to accommodate different needs, ranging from a few days for structural managers to three to 6 months for specialisation in fields like medicine or public health. Integration into specialisation programmes is a viable approach when mutual recognition of segments of professional training can be agreed between those responsible in different scientific and professional societies.

Regarding the economic feasibility of these proposals, there are three aspects: (1) costs to individual practitioners undertaking the training; (2) costs to employers and professional societies currently responsible for funding training programmes; (3) costs of implementing ecologically sustainable options for human activity when such options emerge from the work of practitioners and cost more than the alternative. There is a close relation between these aspects, so that addressing one will facilitate addressing the others. For example, if an employer provides a position with a clear role in EPH, the employer would bear the cost and then the individual could shape their own training whilst employed, making it viable for their own personal career. Decision analysis has aided the reallocation of funding to public health objectives in the case of health services, and in principle such methods could be applied to activities that produce climate and other environmental change with a known effect on health.

### 4.4 Leadership and governance

The scope of EPH across health and non-health sectors indicates that a single source of leadership is not realistic or fruitful. Leadership and governance in this enterprise are inspired by the concept of the conductor and the orchestra. Sharing of fundamental values underlying movement towards ecological sustainability represents the surest foundation for several ‘orchestras and conductors’ to work in harmony. Accordingly, governance, which refers to the tangible framework and operational activities, facilitates the implementation of EPH through collaborative efforts across multiple sectors (transdisciplinary) requiring intersectoral collaborations. For example, when nature-based solutions in agroforestry are implemented with a view to improve planetary health and human health as part of that, it was appropriate for agronomy and forestry specialists to assume leadership (74). Conversely, when an effort was made to characterise the impacts on health and sustainability of alternative choices in pollution-generating activities in urban areas, the leadership was taken by specialists in engineering with input of natural scientists and public health professionals (75).

#### 4.4.1 Role of policy makers

Policy makers can facilitate development and application of promising new approaches, in particular when they permit experimentation in key sectors of the economy at least in a few dedicated geographic areas, to support design and testing of bold proof of concept activities. This would provide an element of dynamic exploration and selection of the most effective solutions alongside policies to support system-wide changes, such as re-design of building codes and agroforestry practises towards ecological sustainability, or introduction of a national skilling wage. Modes of inclusion and dialogue between agencies responsible and groups impacted responsible for or promoting alternative solutions may be reviewed regularly as part of governance arrangements. In any case, a policy framework that can support the new EPH practices would rest on cross-ministry coordination, to clarify that a ministry or department responsible for production of emissions or pollution has a responsibility for EPH alongside the Ministry of Health and the Ministry of Education. This could be as simple as a cross-departmental group with this function or be an element of an integrated technical agency such as in the case of the RIVM in the Netherlands, a multidisciplinary agency funded by four different ministries.

#### 4.4.2 Role of public health practitioners

Health practitioners can play a role in creating sustainable communities by integrating health and social well-being data into decision-making across various sectors. To provide valuable health information for decision-makers outside the health sector, EPH functions are crucial. It may be beneficial for EPH to be led by experts experienced in considering population-wide health effects within specific cultural and historical contexts. They may be invited to act as ‘conductors’ of EPH, offering feedback to decision-makers in other sectors in a transparent manner. This transparency fosters consensus on recommended interventions. For instance, Turin engaged local communities in siting waste treatment facilities through a ‘deliberative democracy process’. Protection within the public health economy is necessary for sustained employment and independence from healthcare sector reforms (76).

This can also refer back to ethics. If intersectoral inclusiveness in informing EPH practice, ethics and legal agreements (or mandates) can be the mechanisms to ensure a running infrastructure.

#### 4.4.3 Obstacles and ways to address them

Obstacles and objections that can be expected in this area are recognised, and concern cultural/disciplinary and institutional barriers, academic competition, and economic/financial resources. The experiences conducted so far highlight the value of moving from agreements in principle to the role of practical arrangements, such as joint supervision by staff in separate organisations of projects by practitioners, secondments with tasks according to a previously agreed joint agenda, professional doctorates where the student is embedded in an organisation where their development of new knowledge is co-designed by business supervisors alongside researchers in academia, and immediately used in the business operations. Such arrangements would support individual career progression whilst also facilitating development of new institutional and inter-agency functions and capacity. The latter would be enhanced by active recognition of the macro-areas or settings, such as school, hospital, shipyard, food production facility as well as the broad function such as data collation, development of guidelines, safety protocol implementation, comparison with operation in similar districts, communication and prevention services. Any specific activity would need to consider a specific setting and function and may benefit from awareness of its implications for environmental public health. Overall, the experiences reviewed confirm that in many cases detailed actionable steps to implementation may be identified that allow institutional and economic barriers as well as academic competition to be overcome, rendering the activity feasible.

In sum, these arrangements would enable any output from EPH to be co-designed and delivered effectively to decision makers and those who advocate certain interventions or courses of action. It would be the responsibility of these non-health sector roles to reflect, question, amplify the conclusions and recommendation with explicit processes that would be specific to each sector.

### 4.5 What should the new practitioner look like?

Various professionals from public and private sectors (health, environment, architecture, etc.) have been identified to contribute to ecologically sustainable health and well-being. Each profession may need to review the design of the competencies of the new Environmental Public Health Practitioner. These practitioners will operate at different levels, from early career to decision-makers. The research component of their professional profile will vary depending on whether they work in academic or service/professional settings in which research is conducted. A European Commission-funded project (DG SANTE) established a network for environmental public health specialist training (77). This initiative identified various approaches within European countries regarding profiles, university courses, training, and registration requirements for health specialists in environmental expertise. Similar efforts exist in other continents. Common elements include the importance of social and natural sciences, epidemiology, and toxicology as foundational knowledge for environmental public health specialists (52, 54). A similar approach could be applied to biologists, geologists, sociologists, architects, and other disciplines involved in environmental public health. Considering the importance of different contexts (e.g. historic, religious, cultural) that affect all sectors of human decision making, the practitioner may benefit from referring to this context when declaring the values underlying the design, analysis, and interpretation of any EPH task. Statements are available from a variety of cultural and religious leaders (78).

It is important for public health agencies to strengthen the visibility and legitimacy of Community-Based Participatory Research (CBPR) approach. This approach will help to enhance the credibility of public health practitioners and improve the preparedness activities of public health boards (79). As mentioned earlier, this approach must be based on a clear and authoritative scientific basis.

To address the needs of such varied collection of practitioners and their interaction and co-operation, a few examples of training scenarios are provided below:

#### 4.5.1 Training integration amongst family medicine practitioners and prevention bodies

An example of training experience in another speciality is clinicians’ experience in preventive services. If a General Practitioner (GP) in training can attend an internship in the specialisation course in Public Health on topics that are part of the training curriculum (for example, organisation, epidemiology, health promotion…), this would allow the student to know the purposes, methods and possibilities of integration with GPs activity.

This training exchange is to be seen as a training enrichment both for individual practitioners and the departments they are attending. The positive result is the training of practitioners to be better able to develop and operate in inter-agency collaborations.

#### 4.5.2 Training integration amongst researchers—prevention officials

An integrated training approach involves exchanges between research institutes, especially epidemiological ones, and specialists in hygiene and preventive medicine. This approach should also include young researchers from various disciplines gaining experience in prevention services. These exchanges allow research and public service institutions to benefit from each other’s expertise and contribute to different projects, promoting mutual learning.

#### 4.5.3 Training integration amongst environmental protection agencies and prevention facilities staff

If such a model could be fruitful for primary prevention in general, it might be even more in the case of environmental prevention. The agencies could ‘exchange’ trainees in the health sector and sectors other than health. In this case, the benefits could be even more significant. It would be a training experience for public health specialists in epidemiology services or Environmental Protection Agencies (EPAs) and, vice versa, by EPA workers with degrees in physics, chemistry, geography, environmental science or other disciplines employed as environmental specialists in an epidemiological research institute or an epidemiology or environmental prevention service, or in a public health laboratory, or food hygiene service, or a veterinary service of a health agency.

The examples provided clearly show that working together leads to familiarity with laws and methods relevant to the professional practice of colleagues active on shared objectives. This familiarity will also take place on a practical level. Hearing a worker on the phone whose context is understood creates the best conditions for creating an effective network aimed at prevention.

#### 4.5.4 From training to practice

In general, the training of officials or consultants with experience in different agencies facilitates the implementation of multi-disciplinary interventions with the skills of various agencies. This integration depends on the awareness that the objectives are achievable only if activities requiring complementary skills are shared.

However, it may be fruitful to arrange accreditation of some roles in environmental public health supported by some legal statements, which recognise that training is linked to a specific integrated training path, which is necessary for the achievement of the speciality, for registration in a professional register and the practice of a legally recognised role in public health.

In addition to training specialists with multi-disciplinary sensitivity and direct knowledge of practitioners ‘in the other field’, it also is advisable to consider a training course of the prevention worker’s career. Placement in other sectors in the post-speciality years or the preparatory phase to managerial positions in public health services or EPAs is essential to achieve multi-disciplinary and inter-institutional communication skills.

Such knowledge sharing might be helpful also to raise awareness of steps needed for sustainable development and to reduce the environmental footprint of health services.

## 5 EPH organisational synergies for education and training

There is a traditional distinction between ‘education’, the development of fundamental cultural and scientific knowledge, and ‘training’, referred to concepts, skills and competencies that constitute the ability to apply knowledge to reality as part of professional or practical roles in society. The tasks of providing education and training even though closely related, are distinct and have several differences; a key one is that training is focused chiefly to ‘practitioners’, people who are already employed and who would like to develop their role, whereas education is mainly focused on ‘students’ who may or may not go on to be professionally involved in applying their knowledge.

The following comments and proposals are addressed to different targets with different objectives, addressing various challenges and modes of action.

The WHO is developing different sets of environmental health tools and training materials for health professionals, such as the air pollution and health training toolkit. A mapping of other air pollution and health training opportunities has also been published (80), presenting courses from different geographical regions whilst providing some good practices for creating new training programmes. WHO has developed various products to educate healthcare professionals on climate change, children’s health, environmental risk factors, and more. They have developed a comprehensive collection of WHO and UN guidance for creating healthier environments, consisting of 500 actions and interventions. This resource is valuable for decision-makers including mayors, public health officers, and ministry staff involved in health and environmental matters (81).

The World Organisation of National Colleges, Academies and Academic Associations of General Practitioners/Family Physicians, the World Organisation of Family Doctors (WONCA), and Environment and Telessaúderes-UFRGS launched the Planetary Health course for Primary Care. With a focus on clinical practice and the reality of health professionals, the course is designed to introduce family doctors and other primary health care professionals and students to planetary health; and to inspire and guide them to educate others or become advocates in various ways (82, 83).

The Global Consortium on Climate and Health Education (GCCHE) was created at Columbia University in 2017. With the aim to develop core competencies for climate and health education and equip healthcare professionals worldwide with the knowledge and skills to address climate-related health challenges, it has an input from over 300 member schools across 50+ countries. They are central to the Climate Change and Public Health Toolkit by the Association of Schools and Programmes of Public Health (84, 85).

As per the Statement of Planetary Health Principles (87), as detailed in Annex, the *in vivo* Planetary Health group affiliated with the Worldwide Universities Network (WUN) contemplates the following:

‘Advocacy: We should actively promote the increased integration of a planetary health perspective into the education of healthcare professionals. Additionally, we should advocate for early-life education in scientific disciplines that serve two critical purposes: first, to demonstrate the intricate interconnectedness of human life with Earth’s biodiversity and natural systems; and second, to illustrate how individual well-being is intricately linked to our coexistence with fellow humans and other life forms’.

Such discourse is encouraged in the education of caring and teaching professionals (and widely throughout society). Individuals will strive to lead by example, reduce primacy, and encourage unity.

## 6 Conclusion

To support conversion of human societies to ecological sustainability before climate and other environmental change produce impacts that threaten the resilience of social fabric catastrophically, it is important to empower practitioners of all disciplines relevant to environmental public health. Supporting the education of future generations to move in this direction, as well as the current generation of decision makers, can help ensure justifications and plans for the more sustainable options within the available spectrum of the workforce making use of their current roles.

Training and enabling practitioners in multiple disciplines for the EPH task is highly encouraged. Practitioners of health and other disciplines may play a role within consortia directed at the overall goal of practical re-orientation of activities with human health impacts and related decision making.

This paper provides a reflection on the overall path of environmental health prevention training and education, focusing on conceptual frameworks of reference that can inform the overall perspective for implementing environmental public health on the ground. A companion paper (86) summarises some experiences and proposals from around the world. These confirm that the call has already been heard and produced several results in the real world; hence, the companion paper presents recommendations for those who arrange training activities in this field. The main point that has been raised is the need for and feasibility of integration, and re-orientation of current practice by on-the-job training inspired by experiences already completed, as well as influence of future practice by re-directed educational frameworks.

## Author contributions

GL: Conceptualization, Supervision, Writing – original draft, Writing – review & editing, Formal analysis. AZ: Conceptualization, Supervision, Writing – original draft, Writing – review & editing, Formal analysis, Funding acquisition. MA: Writing – review & editing. CB: Writing – review & editing. HC: Writing – review & editing. RD-D: Writing – review & editing. RE: Conceptualization, Writing – review & editing. NG: Writing – review & editing. OG: Writing – review & editing. WH: Writing – review & editing. PJ: Conceptualization, Writing – review & editing. EK: Writing – review & editing. PMO: Writing – review & editing. JP: Writing – review & editing. PH: Writing – review & editing. ER: Writing – review & editing. MS: Writing – review & editing. JS: Writing – review & editing. CS: Writing – review & editing. SV: Writing – review & editing. FY: Writing – review & editing. PL: Conceptualization, Formal analysis, Supervision, Writing – original draft, Writing – review & editing.

## Footnotes

^1^Adopted by the General Assembly of the United Nations in 2015, the 17 “Sustainable Development Goals” (SDGs) are the layout to achieve a better and more sustainable world for all by 2030. These goals are a call for action to address a series of global challenges, such as: poverty, inequality, climate, environmental degradation, and justice Essentially for sustainable development to be achieved, it is crucial to harmonise three core elements: economic growth, social inclusion and environmental protection. These elements are interconnected, and all are crucial for the well–being of individuals and societies. From an EPH point of view, all 17 SDGs are different facets of an integrated and systematic approach towards a sustainable and healthy planet.

^2^https://neighbourhood-enlargement.ec.europa.eu/funding-and-technical-assistance/twinning_en

## Annex

**Glossary**

**Capacity-building:** the capacity of individuals and groups to solve problems requires more than training—it also requires networks, supports, and infrastructure. Thus the term encompasses much more than ‘training’ to include team-building, communication skills, and networks (88).

**Competence/Skill:** ‘competencies’ and ‘skills’ are not interchangeable terms. Competencies integrate knowledge, skills, values, and attitudes for effective performance. Skills, on the other hand, are specific abilities that lead to predetermined results in a particular setting, categorised as technical or interpersonal. (‘soft’ skills) (89).

**Community Health:** is a major field of study within the medical and clinical sciences which focuses on the maintenance, protection, and improvement of the health status of population groups and communities (90). The WHO defines community health as environmental, social, and economic resources to sustain emotional and physical wellbeing amongst people in ways that advance their aspirations and satisfy their needs in their unique environment (91).

**Eco-health:** is a field of research, education, and practice that adopts systems approaches to promote the health of people, animals, and ecosystems in the context of social and ecological interactions (92).

**Ecological public health:** focuses on interactions, with one strand focusing on the biological world—in concerns about increasing strains on biodiversity or antimicrobial resistance, for example. Another strand centres on material issues such as links between industrial pollution, energy use and toxicity, and the impact on human species and nature. The advantage of ecological thinking is that it theorises complexity, a key feature facing modern conceptions of health (93).

**Ecosystem health:** Not to be confused with Ecological health or Environmental health Ecosystem health is a metaphor used to describe the condition of an ecosystem. This term is often used in portraying the state of ecosystems worldwide and in conservation and management. For example, scientific journals and the UN often use the terms planetary and ecosystem health, such as the recent journal The Lancet Planetary Health (94).

**Environmental health:** Clean air, stable climate, adequate water, sanitation and hygiene, safe use of chemicals, protection from radiation, healthy and safe workplaces, sound agricultural practices, health-supportive cities and built environments, and a preserved nature are all prerequisites for good health (95).

**Environmental Justice:** is the fair treatment and meaningful involvement of all people regardless of race, colour, national origin, or income with respect to the development, implementation and enforcement of environmental laws, regulations and policies (96).

**Environmental Public Health:** can be defined as ‘The science and art of preventing disease, prolonging life, and promoting health, where environmental risks are an important factor, through the organised efforts of society’ (97). It addresses aspects of health that are determined by interactions with the environment and occurs on many scales: genetic, cellular, individual, family, community, regional, national, and global (99).

**Environmental public health tracking/surveillance:** systematic, ongoing collection and analysis of information related to disease and environment, and its dissemination to individuals and institutions (99).

**Global Health:** The ‘global in global health has in practice referred to the reach of a small set of non-state actors—non-governmental organisations, pharmaceutical companies, philanthropies and universities—capable of defining new health agendas for the planet. The peculiar epistemology of global health emerged in the post-Cold War period from the widely shared belief that the transnational nature of contemporary threats to health—the propagation of infections through air travel, or the rise of chronic diseases associated with trade liberalisation and multinational corporations—could not be addressed through the old international health system, built around nation-states, but required new global solutions able to work across political and geographical borders (100).

**Health Equity:** Equity is the absence of unfair, avoidable or remediable differences among groups of people, whether those groups are defined socially, economically, demographically, or geographically or by other dimensions of inequality (e.g. sex, gender, ethnicity, disability, or sexual orientation). Health is a fundamental human right. Health equity is achieved when everyone can attain their full potential for health and well-being. (https://www.who.int/health-topics/health-equity#tab=tab_1).

**Health Inequalities:** are unfair and avoidable differences in health across the population, and between different groups within society. (https://www.england.nhs.uk/about/equality/equality-hub/national-healthcare-inequalities-improvement-programme/what-are-healthcare-inequalities/).

**One Health:** is an integrated, unifying approach that aims to sustainably balance and optimise the health of people, animals and ecosystems. It recognises the health of humans, domestic and wild animals, plants, and the wider environment (including ecosystems) are closely linked and interdependent. The approach mobilises multiple sectors, disciplines and communities at varying levels of society to work together to foster wellbeing and tackle threats to health and ecosystems whilst addressing the collective need for clean water, energy and air, safe and nutritious food, taking action on climate change, and contributing to sustainable development (101).

**Planetary health:** The definition published by The Lancet: ‘the achievement of the highest attainable standard of health, wellbeing, and equity worldwide through judicious attention to the human systems—political, economic, and social -that shape the future of humanity and the Earth’s natural systems that define the safe environmental limits within which humanity can flourish. Simply put, planetary health is the health of human civilisation and the state of the natural systems on which it depends’ (102). Planetary health is the interdependence of all ecosystems, natural and human-made, promoting health equity and wellness. It requires integrated approaches, breaking down conventional boundaries to foster science and cultural collaborations (87).

**Primary health care:** is a whole-of-society approach to health and wellbeing centred on the needs and preferences of individuals, families and communities. It addresses the broader determinants of health and focuses on the comprehensive and interrelated aspects of physical, mental and social health and wellbeing. It provides whole-person care for health needs throughout the lifespan, not just for a set of specific diseases. Primary health care ensures people receive comprehensive care—ranging from promotion and prevention to treatment, rehabilitation and palliative care—as close as feasible to people’s everyday environment (103).

**Primary and Community health care:** could play in managing health emergencies, addressing key environmental and social aspects, and assisting in designing the interventions to control them. P&CHC is important locally and globally. Societies are ageing, health spending is rising, and it is imperative to redesign health systems that contribute to community health by addressing environmental factors (21).
